# A Relative Bioavailability Study of Two Misoprostol Formulations Following a Single Oral or Sublingual Administration

**DOI:** 10.3389/fphar.2020.00050

**Published:** 2020-02-12

**Authors:** Mahdi Amini, Margareta Reis, Dag Wide-Swensson

**Affiliations:** ^1^Department of Obstetrics and Gynecology, Skåne University Hospital, Lund, Sweden; ^2^Department of Clinical Chemistry and Pharmacology, Skåne University Hospital, Lund, Sweden

**Keywords:** human, pregnancy, induction of labor, misoprostol, pharmacokinetics

## Abstract

**Introduction:**

Misoprostol (Cytotec) was primarily made for treating gastric ulcers. However today it is mostly used for abortion, treating postpartum hemorrhage, and for induction of labor. The tablet contains 200 µg of misoprostol, yet the dosages used for induction of labor are much smaller (25–50 µg), leading to uncertainty of dosage in daily use.

**Aim:**

To evaluate and compare the relative bioavailability of two misoprostol products (Angusta 25 µg and Cytotec 200 µg tablets) administered orally or sublingually given in a daily clinical setting to women admitted for induction of labor at term.

**Methods:**

Women carrying a live, singleton fetus in a cephalic position and with a gestational age between 259 and 296 days were included. Blood samples were collected at 0, 5, 10, 20, 30, 40, 50, 75, 100, 120, 180, and 240 minutes. A serum analytical assay was performed and pharmacokinetic parameters were calculated. Patients were assigned to one of three groups.

**Results:**

A total of 72 patients were included. No significant differences demographic characteristics were found. The ratios for AUC, AUC (_0−t_), and C_max_ were similar in all three groups, but CI-values were outside the required 80–125%. Sublingual administration yielded a 20–30% higher bioavailability and a 50% higher C_max_ than compared to the oral route.

**Conclusion:**

The relative bioavailability between Angusta and Cytotec could not be confirmed as being equal at the 25 µg or 50 µg level because the 90% CI-values when comparing the ratios for AUC, AUC_(0−t)_, and C_max_ were wider than accepted. The reason for this could be the real-life, non-standardized circumstances in which the study was conducted. Sublingual administration seems to have higher bioavailability than oral administration. More studies are needed to ascertain an optimal dosage regime balancing both safety and efficacy for mother and child.

**Clinical Trial Registration:**

www.ClinicalTrials.gov, identifier NCT02516631.

## Introduction

Bioquivalence of generic and branded products is based on two key pharmacokinetic (PK) measures: area under the concentration– time curve (AUC) and maximal concentration (C_max_) (US Food and Drug Administration). Differences in up to 20% of drug bioavailability is concluded not to be of clinical significance. If the respective 90% CI of the ratios of the generic to the branded compound for the AUC and C_max_ fall within 80% to 125%, then these products are considered to be bioequivalent (Bioequivalence Task Force, 1988; Center for Drug Evaluation and Research; US Food and Drug Administration, 1992; Center for Drug Evaluation and Research; US Food and Drug Administration, 2001; Galgatte et al., 2014). This range was established from a FDA Bioequivalence Hearing and based on both statistical analysis and expert opinion (Bioequivalence Task Force, 1988). It has been under debate that this range might be too broad, yet most bioequivalent products actually show a smaller difference than the required 20% (Henderson and Esham, 2001; Davit et al., 2009).

Induction of labor, IOL is one of the most common obstetrical procedures taking place at obstetrical units. The incidence is increasing worldwide every year (Rayburn and Zhang, 2002). The induction rate varies from 8 to 24% in Swedish hospitals 2016. At most Swedish clinics the induction rate has doubled during the last decade (The National Board of Health and Welfare in Sweden, 2015). The methods of IOL are several, both mechanic and pharmacological where prostaglandins are the most commonly used drug (Mozurkewich et al., 2011).

Misoprostol is a prostaglandin E_1_ analog which was manufactured for treatment of gastric ulcers. It is a pro drug metabolized through rapid de-esterificiation into the active metabolite misoprostol acid, MPA. (Schoenhard et al., 1985). Further, it is used world-wide off label for termination of pregnancy, treatment of postpartum bleeding, and also for IOL (Alfirevic et al., 2000; Blum et al., 2007). Misoprostol can be administered in various routes and is economically favorable in daily clinical use. The most common routes are the oral and vaginal routes, but even sublingual, buccal, and rectal routes are also being used (Tang et al., 2007). However only oral and vaginal application is approved by the regulatory agencies. Sublingual administration is of particular interest as it bypasses the first-pass metabolism by the liver (källa) (Tang et al., 2007).

Several studies have investigated the pharmacokinetic properties of misoprostol during termination of pregnancy (Tang et al., 2002; Tang and Ho, 2006; Tang et al., 2009), yet only few studies have been conducted where the pharmacokinetics of misoprostol given to pregnant women at term for IOL are studied. The optimal route of administration and the optimal dose of prostaglandins is a subject of continuous research and still yet to be found.

The IOL regime used at our clinic consists of a misoprostol tablet (Cytotec^®^, Pfizer, Germany) being dissolved in water and the patient given a specific amount of that solution every other hour or a higher dose given every 4 hours until adequate cervical ripening is achieved (Hofmeyr et al., 2001a; Hofmeyr et al., 2001b). Angusta^®^ (Azanta Danmark A/S, CPH, Denmark) is a new tablet containing 25 µg of misoprostol intended for oral use for IOL.

The primary aim of this study is to evaluate the relative bioavailability by comparing the pharmacokinetic properties of these two misoprostol products administered orally or sublingually.

## Subjects and Methods

### Study Design and Drug Administration

This is an open-label, naturalistic, randomized, single-dose, comparative bioavailability study conducted at the Department of Obstetrics and Gynecology, Skåne University Hospital in Lund and Malmö, Sweden during the time period 2014–2016. A total of 72 patients were randomized to three groups.

### Study Population

Inclusion criteria were pregnant women equal to or above 18 years of age eligible for induction of labor carrying a live, singleton fetus in a cephalic position with a gestational age of 37 + 0 weeks to 42 + 0 weeks.

Women with known allergy to misoprostol or other prostaglandins, prior uterine scar, dead or anomalous fetus, and women with known liver or renal dysfunction or multiple pregnancy were not eligible for enrollment.

### Treatment Groups and Treatment Arms

Oral 25 µg (Group A):

1. One tablet of Angusta^®^ (25 µg)
2. Twenty-five ml of Cytotec solution (25 µg)

Oral 50 µg (Group B):

1. Two tablets of Angusta^®^ 25 µg (total 50 µg)
2. 50 ml of Cytotec solution (50 µg)

Sublingual 50 µg (Group C):

1. Two tablets of Angusta^®^ (total dose of 50 µg).
2. One-fourth of a tablet of Cytotec^®^ (50 µg), given sublingually. A tablet of 200 µg is cut with a tablet cutter into four equal pieces. Patients is instructed not swallow for a period of 5 minutes.

The randomization procedure was blinded to avoid investigator bias in allocation of treatment to the subjects. Blinding of the treatment was not possible as the comparator product (Cytotec^®^) was administered in accordance with current clinical practice. Cytotec is available as a 200 µg tablet, necessitating cutting of tablets or dissolving tablets in water before use. The test product (Angusta^®^) is a 25 µg tablet taken whole without further preparation. The oral misoprostol (Cytotec) solution was prepared by dissolving a 200 µg tablet in 200 ml of water, yielding 1 µg/ml.

### Sampling and Medical Supervision

Venous blood (6 ml) was collected into a Vacuette tube through an indwelling cannula placed in one of the prominent veins of the forearm or wrist. For patients enrolled in group A, the blood samples were collected at 0, 5, 10, 20, 30, 40, 50, 75, 100, and 120 minutes after the administration of misoprostol. For patients enrolled in group B or C additional blood samples were taken at 180 and 240 minutes.

After inversion 6 to 8 times, the blood sample tubes were placed on ice. The samples were then centrifuged to obtain the plasma for 10 minutes at 1500g at nominal 4^°^C. For each sample, the separated plasma was transferred into two 1.5 ml Nunc Cryo plasma tubes (primary and back-up) and stored within 2 hours of collection at approximately −20°C. The blood samples were sent in cryoboxes to York Bioanalytical solutions, England, UK for analysis.

The subjects were under continuous medical supervision at the labor ward during the study. Tolerability was evaluated by monitoring adverse events and by physical examinations when needed.

### Drug Analysis Method or Serum Analytical Assay

A solid phase extraction and liquid chromatography tandem mass spectrometry method has been developed and validated for the determination of misoprostol acid in human plasma in the range of 5–500 pg/ml (i.e. the lower limit of quantification = 5 pg/ml). The validation of the assay was conducted according to International Conference on Harmonisation (ICH) requirements.

### Pharmacokinetics and Bioavailability Analysis

The primary outcome measures of the study were calculating the following pharmacokinetic parameters:
AUC_0−t_: Area under the plasma concentration vs. time curve from the first time point (t = 0) to the last measured concentration.
AUC: The area under the serum concentration curve from zero to infinity.
C_max_: Maximum plasma concentration; taken directly from measured values.
T_max_: Time to maximum plasma concentration; taken directly from measured values.
t_½_: Elimination half-life calculated as 0.693/lambda_z_.

Means (geometric and arithmetic), standard deviation, coefficient of variance, median, maximum and minimum were calculated for all pharmacokinetic parameters of all the analytes. Ln-transformed data of C_max_, AUC_0−t_, and AUC was used when calculating geometric mean and least square ratio.

The 90% CI of the ratio of the geometric means of test/comparator for C_max_, AUC_0−t_, and AUC should be within 80% to 125% to claim bioequivalence. This will be calculated using Ln-transformed data of C_max_, AUC_0−t_, and if possible also AUC.

Relative bioavailability between oral and sublingual administration (group B and C) was calculated; preferably from AUC and otherwise from AUC_0−t_. Plasma concentration profiles were subjected to non-compartmental pharmacokinetic analysis using validated PC-based software, WinNonlin v. 6.3 (Pharsight Corporation, Mountain View, CA, USA). The WinNonlin model used was no. 200 (extravascular administration) and PK parameters were assessed. Test for bioequivalence (BE) was performed using the same WinNonlin software for analysis of parallel design. The assessment was performed on dose normalized (divided by dose/kg) ln-transformed AUC, AUC_(0−t)_, and C_max_. Half-lives were compared by t-test on λ_z_ using GraphPad Prism3 (GraphPad Software, Inc., 10855 Sorrento Valley Road #203, San Diego, CA 92121 USA).

Distributions of continuous variables were subject to the Kolmogorov-Smirnov test for normality. The chi-square test and Fisher's exact test were used for comparing categorical variables. For normally distributed variables the unpaired student t-test was used. Non-normally distributed variables were analyzed by the Mann-Whitney U test. A p-value of <0.05 was considered statistically significant.

Oral and written information were given and all patients signed an informed consent. The study was approved by the ethical review board of Lund and Swedish Medical Products Agency.

## Results

### Study Population

**Figure 1:**
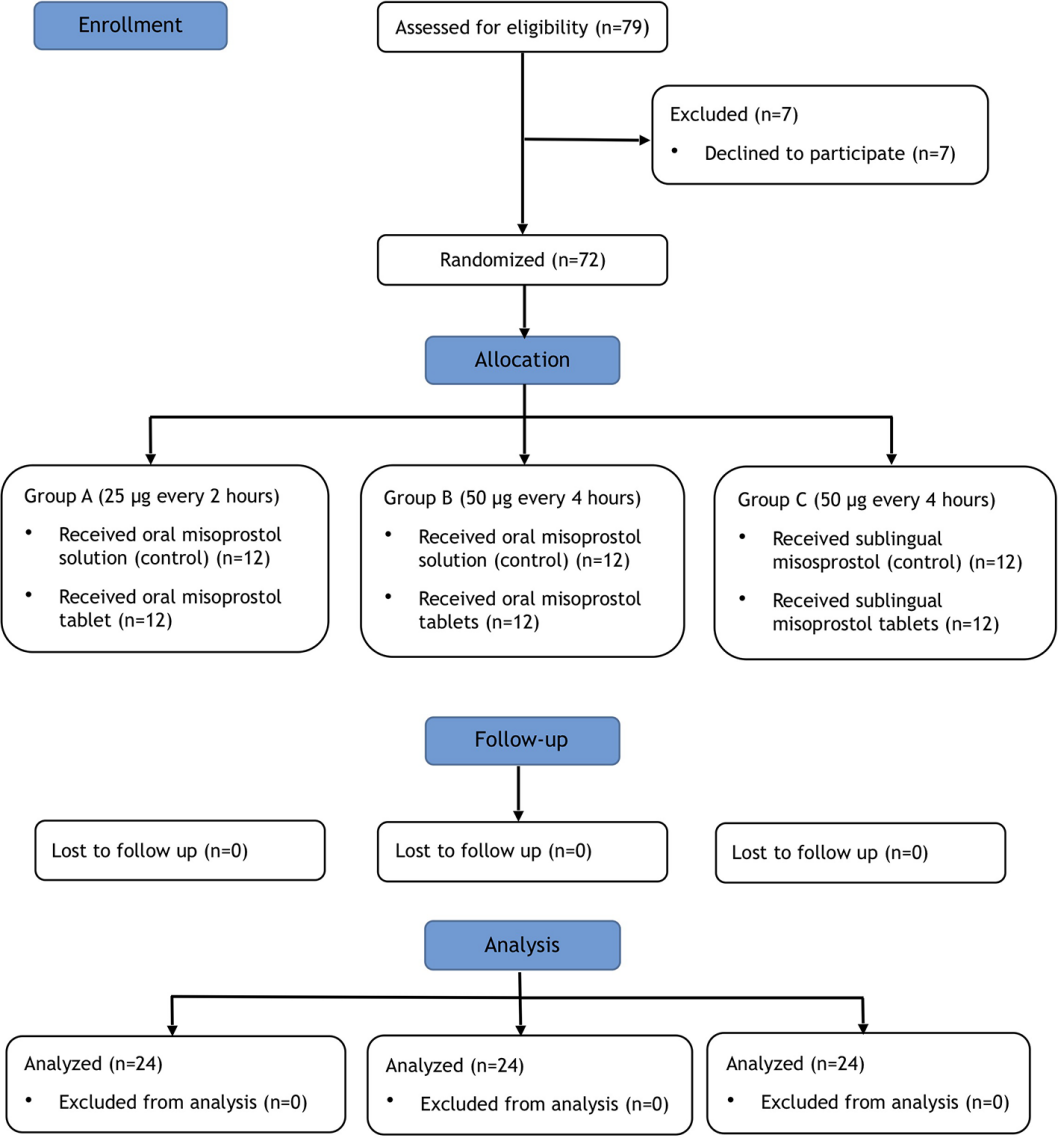
CONSORT flow chart of included patients in the study.

A total of 72 patients were recruited (**Figure 1**). Demographic characteristics are listed in **Table 1**. There were no significant differences in the groups with regards to demographic parameters.

**Table 1:** Demographics characteristics.

	Group A (oral)		Group B (oral)		Group C (sublingual)		Total
	Angusta n = 12	Cytotec n = 12	Angusta n = 12	Cytotec n = 12	Angusta n = 12	Cytotec n = 12	n = 72
**Age (years)**	34.8 (5.57)	32.3 (6.08)	31.5 (2.81)	32.8 (4.43)	33.0 (4.35)	32.8 (5.56)	32.9 (4.88)
**Weight (kg)**	90.9 (14.22)	81.1 (11.80)	79.4 (9.23)	81.3 (13.08)	84.0 (13.74)	94.8 (18.57)	84.2 (13.68)
**BMI (kg/m^2^)**	31.6 (4.40)	29.7 (5.21)	28.9 (3.09)	28.6 (4.68)	30.1 (4.96)	34.2 (5.69)	30.4 (4.48)

Values are mean ± SD, BMI body mass index.

### Pharmacokinetic Properties

Bioavailability testing was based on ln-transformed AUC, AUC_(0−t)_, and C_max_ (**Table 2**).

**Table 2:** Pharmacokinetic parameters.

	Group A (oral)		Group B (oral)		Group C (sublingual)	
	Angusta 25 μg	Cytotec 25 μg	Angusta 50 μg	Cytotec 50 μg	Angusta 50 μg	Cytotec 50 μg
**C_max_ (pg/mL)**	26.64 (22.48)	37.58 (13.53)	57.87 (41.28)	53.97 (23.79)	84.04 (48.77)	100.66 (60.36)
AUC_(0−t)_ (h*pg/mL)	18.22 (10.69)	26.30 (9.90)	53.79 (20.95)	55.02 (28.80)	68.95 (27.83)	83.65 (40.54)
AUC (h*pg/mL)	26.78 (15.43)	35.61 (13.18)	64.05 (22.80)	66.63 (33.22)	76.51 (27.36)	91.60 (40.58)
**t_max_ (hours)**	0.47 (0.54)	0.27 (0.24)	0.46 (0.42)	0.23 (0.14)	0.38 (0.13)	0.27 (0.11)
**t_1/2_ (hours)**	0.82 (0.58)	0.76 (0.47)	0.74 (0.51)	1.05 (0.86)	0.67 (0.20)	0.78 (0.39)
**Ratio (%)** Angusta/Cytotec (90% CI)*	78.4 (56.7–108.5) *p = 0.5528*		104.5 (74.0–147.5) *p = 0.2897*		87.0 (64.3–117.7) *p = 0.3456*	
**Ratio (%)** Angusta/Cytotec (90% CI)✝	66.3 (43.8–100.3) *p = 0.7866*		104.0 (70.9–152.5) *p = 0.3352*		89.8 (58.1–138.8) *p = 0.4295*	

Values are mean ± SD, p-values are calculated based on equality between formulations.

*Based on AUC (h*pg/mL).

✝Based on C_max_ (pg/mL).

#### Group A

**Figure 2:**
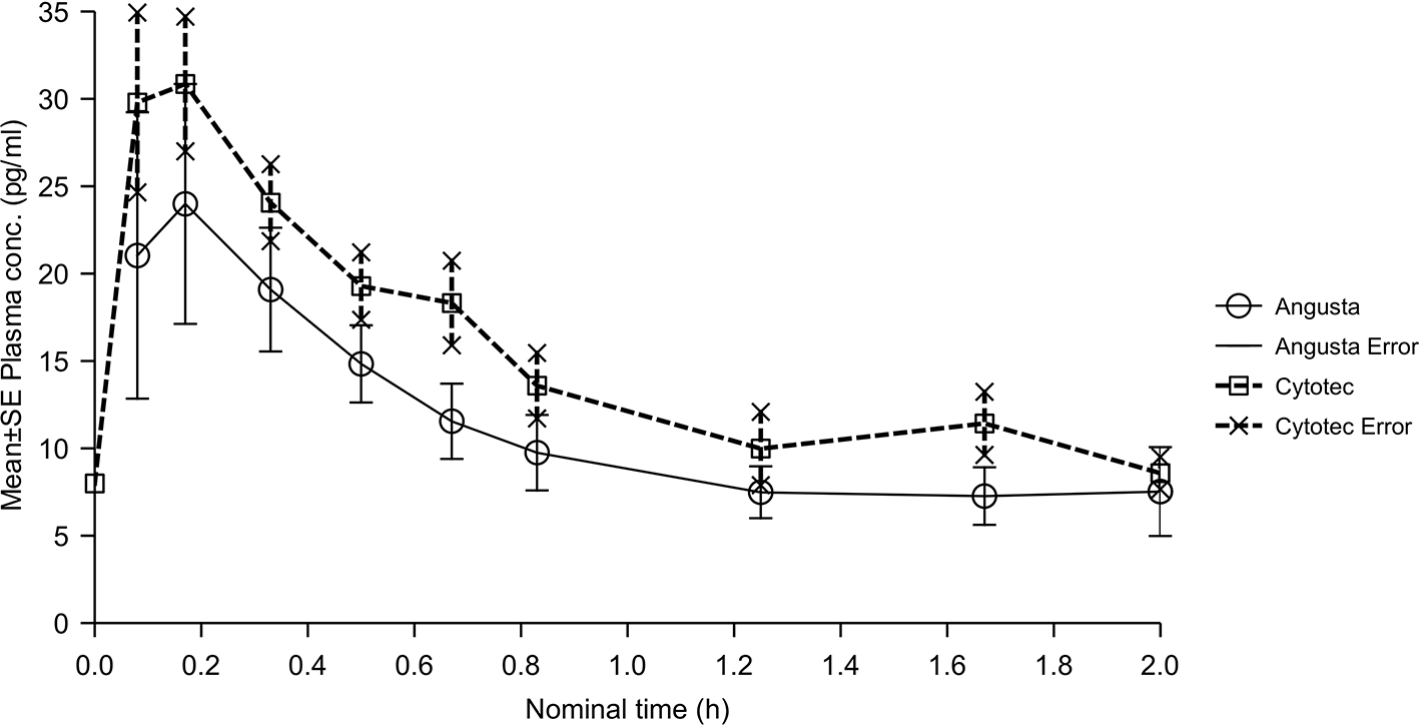
Mean plasma concentration-time profiles of misoprostol acid after a single 25 µg dose of Angusta or Cytotec formulation given orally.

The combined mean plasma concentration profiles shown in **Figure 2**. The bioavailability analyses resulted in ratios for AUC and AUC_(0−t)_ that were different and CI values that were not in agreement with the requirement for the relative bioavailability to be equal. Therefore, relative bioavailability between Angusta tablet and Cytotec oral solution could not be confirmed as being equal at the 25 µg dose level. We did not find a statistical difference between the two formulations regarding half-lives, compared by t-test of λ_z_ (p = 0.7586). The time to peak concentration, t_max_ for Angusta and Cytotec were 0.47 hours (SD ± 0.54) and 0.27 hours (SD ± 0.24) respectively.

#### Group B

**Figure 3:**
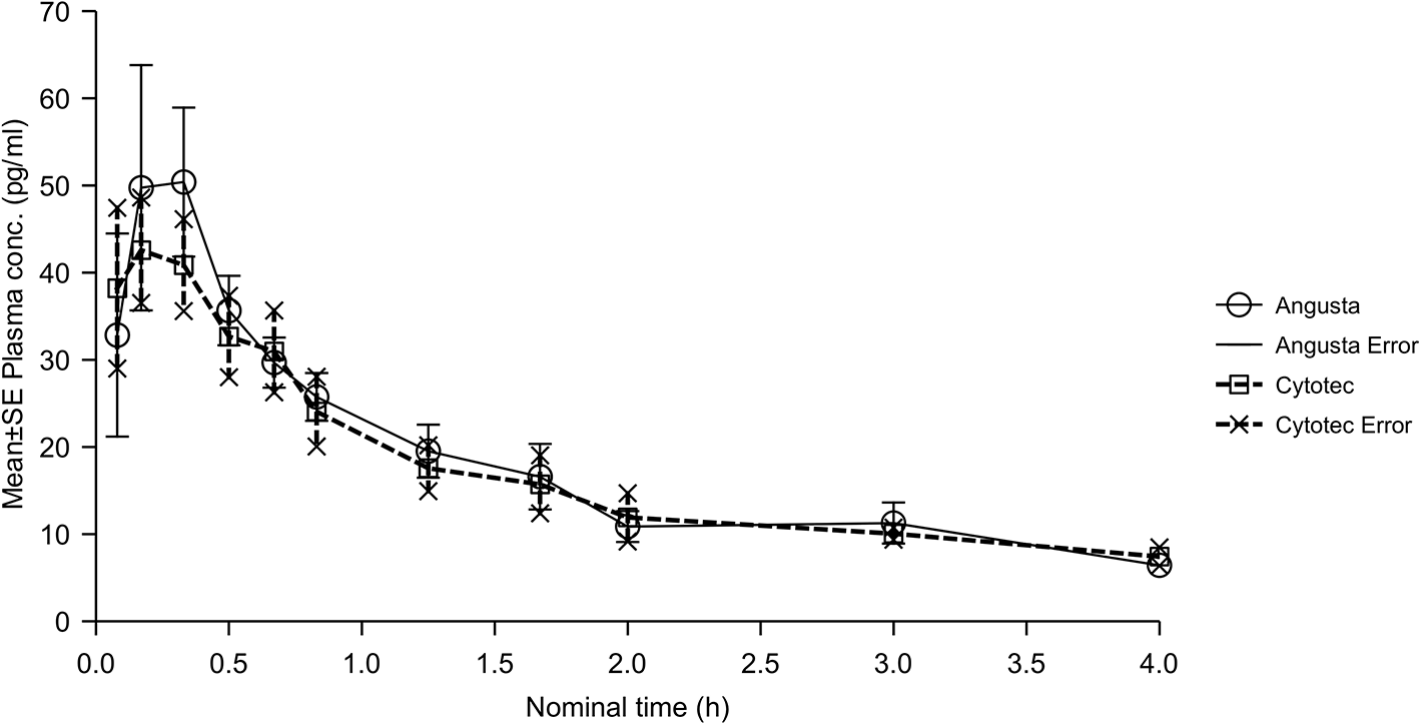
Mean plasma concentration-time profiles of misoprostol acid after a single 50 µg dose of Angusta or Cytotec formulation given orally.

The bioavailability analyses of oral 50 µg misoprostol resulted in ratios for AUC, AUC_(0−t)_, and C_max_ that were quite similar between the two formulations, but the CI-values were not within the required 80–125%. Half-lives, compared by t-test of λ_z_, were not statistically significantly different between the two formulations (p = 0.1695). The combined mean plasma concentration profiles shown in **Figure 3**. T_max_ was 0.46 hours (SD ± 0.42) for Angusta and 0.23 hours (SD ± 0.14) for Cytotec.

#### Group C

**Figure 4:**
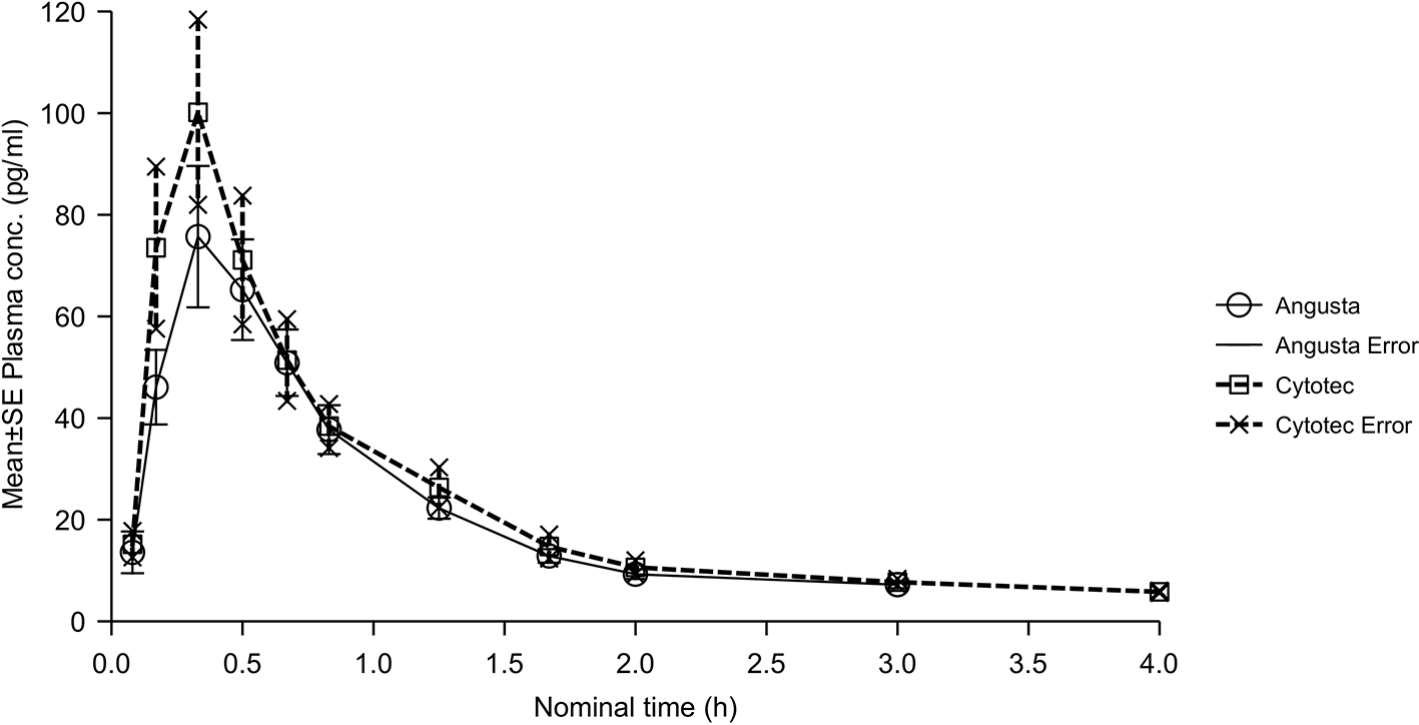
Mean plasma concentration-time profiles of misoprostol acid after a single 50 µg dose of Angusta or Cytotec given sublingually.

In the sublingual 50 µg calculations the ratios for AUC, AUC_(0−t),_ and C_max_ were similar for the two groups, and just below 90%. CI-values were outside the required 80–125% (**Table 2**). The combined mean plasma concentration profiles are shown in **Figure 4**. Half-lives, compared by t-test of λ_z_, were not statistically significantly different between the two formulations (p = 0.0550). T_max_ for Angusta was 0.38 hours (SD ± 0.13) and 0.27 hours (SD ± 0.11) for Cytotec.

## Discussion

In this study, we have compared the bioavailability of two misoprostol formulations in two different doses given through either the oral or sublingual route. The study provides insight into various pharmacokinetic properties of misoprostol used for induction of labor in term pregnant women in a daily clinical setting. Our study was conducted to ascertain whether misoprostol in a specific tablet form (Angusta^®^) could be shown to have the same bioavailability as the dissolved form of misoprostol (Cytotec^®^) often used in IOL.

The primary aim of this study was to evaluate the relative bioavailability by comparing the pharmacokinetic properties of these two misoprostol products administered orally or sublingually. Cutting the Cytotec tablets into four or eight small pieces poses a risk of dosage inaccuracy. The reason for comparing Cytotec to Angusta is that the exact dose of misoprostol in the Angusta tablet overcomes the risk of dosage inaccuracy when Cytotec is cut into four or eight pieces. Correct dosing is crucial to maintain efficacy and safety during IOL and to keep side effects to a minimum.

Several changes in physiology during pregnancy could have affected to the result of our study. Firstly, the increase in progesterone during pregnancy decreases intestinal motility and delays gastric emptying which could, in part explain the differences seen between the oral tablet and solution (Parry et al., 1970).Pregnancy also affects the volume distribution due to a 50% increase in plasma volume and therefore a relative hypoalbuminemia (Costantine, 2014). This in combination with a pregnancy induced increase in renal blood flow could also affect the clearance of active misoprostol acid metabolite. Pregnancy also induces changes in several hepatic enzymes which could explain the differences seen between oral and sublingual route as the sublingual route bypasses the first-pass metabolism by the liver (Koren and Pariente, 2018).

In the oral 25 µg group, bioavailability analyses resulted in ratios for AUC and AUC_(0−t)_ that were different and the CI-values were too wide to be accepted. This might be due to the relatively low dose that was tested and to the non-fasted state of the patients. For the 50 µg oral dose (Group B), bioavailability ratios between Angusta and Cytotec were comparable. However, the 90% CI-values were wider than accepted. The reason for this could be the real-life, non-standardized circumstances in which the study was conducted. Based on the appearance of the mean plasma concentration plot it appears reasonable to consider Angusta bioequivalent to Cytotec. The bioavailability ratios between Angusta and Cytotec in the sublingual group did not contradict the bioavailability of being equal, but even here the CI-values were wider than accepted.

With regards to standardization of misoprostol solution (tablet dissolved in water) the Swedish Medical Agency (SMA) have investigated the patency of the misoprostol solution when given in 20–25 µg doses (Swedish Medical Products Agency, 2012). They found that the mean misoprostol concentration was 19.7 µg (range 19.5 µg–20.3 µg, nominal dose 20 µg) leading to 98.3% of the intended 20 µg dose. A sample from the solution was also analyzed after 3 hours where the misoprostol concentration was 96.9% of the intended dose. No significantly different concentrations were found whether the solution was stirred prior to sampling or not. Since there are no data regarding the patency of the solution after the 3-hour time frame, the SMA recommends discarding any unused solution after administering each dose.

The oral misoprostol solution seems to show a tendency for shorter T_max_ than the Angusta tablet. In a study conducted by Chong et al, patients were given 400 µg misoprostol either as an oral tablet, oral solution, or the vaginal or rectal routes post-partum and the uterine pressure was measured (Chong et al., 2004). The oral solution given had a shorter median onset of action (4.0 minutes, range 2.0–5.0 minutes) compared to misoprostol given as tablets orally (6.0 minutes, range 4.0–10.0 minutes, P = 0.01).

The T_max_ in the oral groups of our study are similar to previous reports by Zieman et al. (T_max_ = 34 minutes) (Zieman et al., 1997) and Tang et al. (T_max_ = 24 minutes) (Tang et al., 2002). The difference in T_max_ could be explained by the fact that the misoprostol already is dissolved in the oral solution, leading to a quicker uptake in the gastrointestinal canal.

We also undertook a bioavailability testing based on ratios of ln-transformed AUC, AUC_(0−t)_, and C_max_ between oral and sublingual Angusta indicated a 20–30% higher relative bioavailability and a 50% higher C_max_ for the sublingual route at the 50 µg dose level (**Table 3**). The same comparison between oral and sublingual Cytotec indicated a 40–55% higher bioavailability and a 70% higher C_max_ for the sublingual route (**Table 4**). These results are in line with a study conducted by Tang et al. where they compared the pharmacokinetics of misoprostol in women undergoing termination of pregnancy (Tang et al., 2002). Another pharmacokinetic study where non-pregnant volunteers were given misoprostol either by oral, sublingual, or buccal route showed a significant increase in bioavailability for the sublingual route compared to the oral route (Frye et al., 2016). A possible explanation for the difference between oral and sublingual administration could be that misoprostol when given by the sublingual route bypasses first-pass metabolism by the liver, leading to higher plasma concentration levels than when compared to the oral route. The relative neutral pH in the oral cavity could also be a factor. Further, when a tablet is administered sublingually the patient is told not to swallow the saliva. However, there might some cases when the patient will swallow their saliva too early. This can lead to that the patient swallowing saliva earlier than required. It is possible that this can explain the difference between the two formulations of misoprostol.

**Table 3:** Oral Angusta 50 µg vs Sublingual Angusta 50 µg.

		Mean ± SD	Sublingual/Oral (90% CI)	p-value*
**AUC** **(h∙pg/ml)**	Sublingual Oral	76.51 ± 27.36 64.05 ± 22.80	119.8 (92.9–154.5)	0.3944
**AUC_(_**_0−t_**_)_ (h∙pg/ml)**	Sublingual Oral	68.95 ± 27.83 53.79 ± 20.95	128.6 (96.9–170.7)	0.5726
**Cmax** **(pg/ml)**	Sublingual Oral	84.04 ± 48.77 57.87 ± 41.28	149.0 (103.4–214.7)	0.7949

*for equality between formulations.

**Table 4:** Oral Cytotec 50 µg vs Sublingual Cytotec 50 µg.

		Mean ± SE	Sublingual/Oral (90% CI)	p-value*
**AUC** **(h∙pg/ml)**	Sublingual Oral	91.60 ± 40.58 66.63 ± 33.22	143.9 (97.5–212.4)	0.7382
**AUC_(_**_0−t_**_)_ (h∙pg/ml)**	Sublingual Oral	83.65 ± 40.54 55.02 ± 28.80	156.8 (104.4–235.8)	0.8307
**Cmax** **(pg/ml)**	Sublingual Oral	100.66 ± 60.36 53.97 ± 23.79	172.6 (109.8–271.2)	0.8873

*for equality between formulations.

Our study shows a large variation in the uptake of misoprostol given in a daily clinical setting. A standard bioavailability study would be carried out as a crossover study and enroll healthy volunteers of roughly the same age and weight, would be performed under settings including standard fasted state, eventually a standard meal and similar (limited) physical activity. Our study reflected the real-life situation under which misoprostol is used and did not fulfill any of these standards. Therefore, if these factors were to be standardized for both groups, we believe that true equal bioavailability could be achieved between these two drugs.

The study was a parallel design; comparison between the same group of subjects treated with both test and comparator treatment was not possible. Since the patients were not fasted they were likely to have had food and drink in varying amounts and quality at different times before administration of study drug. These are conditions that would be expected to influence rate as well as extent of absorption. This could also explain the vast range of variability in the uptake of misoprostol that we encountered during the observational period. A broad range of values could also be found in the sublingual group which should not be as sensitive to the fasting state as the oral cohorts. Further, due to the relatively low doses, plasma concentrations were only measurable for short periods, which would be likely to influence the precision of estimated/calculated PK parameters.

Misoprostol (Cytotec), when given sublingually as 50 µg, is given as a quarter of a 200 µg tablet. Even when using a cutting device it is impossible to get an exact quarter of a tablet. Further, we do not know if the active drug is equally distributed in each tablet which increases the dosage inaccuracy when the tablet is also cut into smaller pieces. Misoprostol is known to be stable at room temperature, but this stability is only preserved if the tablets are still in their alveolus/blister packaging. When handling the 200 µg Cytotec tablets to achieve smaller doses the tablets are also exposed to atmospheric conditions. One study investigated the impact of atmospheric conditions to the tablets and found a 5% decrease in the active ingredient after 48 hours and 10% decrease after one week (Berard et al., 2014). The clinical impact of this misoprostol degradation can be difficult to quantify and currently there are no current studies assessing this matter.

In an optimal situation, to fully understand the pharmacokinetics of a drug administered to pregnant women, one should conduct serial studies during the first, second, and third trimester and even the post-partum period so each woman acts as their own control. To truly ascertain the changes in misoprostol uptake in term pregnant women in our current study, one could conduct a follow-up pharmacokinetic study where the delivered women are given the same dose of misoprostol after a wash-out period, for example 6 weeks post-partum.

Prior to this study, we theorized that there might be some differences in the pharmacokinetics of misoprostol in late pregnancy when compared to early pregnancy, yet the results from our study show comparable pharmacokinetics. To the best of our knowledge, no other study with this large number of patients where the pharmacokinetic properties of misoprostol in term pregnant women undergoing induction of labor have been conducted.

## Conclusion

Relative bioavailability between Angusta and Cytotec could not be confirmed as being the same at the 25 or 50 µg levels. The reasons for this could be due to the relatively low doses given which were only measurable for a short time, the real-life circumstances in which the study was conducted and to the fact that the patients were not fasted.

The sublingual route of misoprostol seems to have more pharmacokinetic appealing properties for term pregnant women undergoing induction of labor, with a shorter T_max_ and a higher AUC and C_max_ than compared to the oral route. The pharmacokinetic properties of misoprostol in term pregnant women do not seem to differ significantly from early pregnant women. Further studies are needed where different doses, dosage intervals, and administration routes depending on the indication for IOL are explored. For the future, we recognize the need for large-scale, randomized, dose-response studies for misoprostol given orally or sublingually for induction of labor.

## Data Availability Statement

The datasets generated for this study are available on request to the corresponding author.

## Ethics Statement

This study was carried out in accordance with the recommendations of the Ethical Review Board of Lund, with written informed consent from all subjects. All subjects gave written informed consent in accordance with the Declaration of Helsinki. The protocol was approved by the Swedish Medical Products Agency.

## Author Contributions

MA and DW-S conceived the idea and carried out the experiment. MA wrote the manuscript with support from DW-S and MR. All authors contributed to the final version of the manuscript.

## Funding

The authors declare that this study received funding from Azanta A/S. The funder had no role in study design, data collection and analysis, decision to publish, nor in preparation of the manuscript.

## Conflict of Interest

The authors declare that the research was conducted in the absence of any commercial or financial relationships that could be construed as a potential conflict of interest.
